# Ireland's "Lusitania"

**Published:** 1918-10-26

**Authors:** 


### Ireland's "Lusitania."

The appalling tragedy of the Royal Mail Steamship *Leinster* sent a thrill of horror round the world, and its occurrence at the moment that Germany's "democratic" rulers are seeking a settlement on "friendly terms" sheds an illuminating light on the mentality of the Hun. For cold-blooded devilry this act of barbarism is unsurpassed. Three torpedoes were fired. The first missed its aim. The second reached its target, and it would have accomplished its design, for the mailboat would have sunk. *But the passengers would have been saved.* The third torpedo struck her amidships, and ensured the death of hundreds of helpless passengers, including many women and children. The *Leinster*, built for speed, was shattered, and she sank immediately. Our Irish correspondent informs us that there were many nurses on board, as well as several doctors. Mrs. Carlyle, recently married, happily among the saved, daughter of Mr. Ferguson, of Dublin, was a nurse at Etaples when the hospital was bombed by the Germans. Sister Mary Teresa Murphy is amongst the saved; she was returning to a Nottingham hospital, having attended her brother's funeral. But there were many lost. Nurse Delia Brennick was on her way to Leeds; two sisters, the Misses Davoren, co. Clare, and (also two sisters) the Misses O'Grady, from the same county, were returning to England after their holidays—all nurses; Nurse O'Shaughnessy, Baggot Street, Dublin. All these were drowned; and this by no means exhausts the list of nurses. Miss Barrett, co. Galway, was a V.A.D. on her way to France, and Miss Anna Maud Barry, also a V.A.D., proceeding to Sutton Veney Military Hospital, are amongst the lost. There were many doctors, including Professor Sir William Henry Thompson, K.B.E., T.C.D.; Dr. Ernest Lee, R.A.M.C., son of a prominent Dublin citizen, returning after a brief holiday to France; Dr. Digby Burns, of Dublin; Dr. J. A. Furlong, St. Louis, Mo. (U.S.A.); Dr. Fennessy, Waterford. It is a ghastly list; and these are not all.

For this act of barbarism there is no excuse. Coming as it does when Germany is suing for peace it reminds the world that this war is being waged against a nation of monsters. The men who struck the *Lusitania* Medal to celebrate the agonised cries of women and children hurled by them into the hungry waves thereby set a seal to their iniquity. The Kaiser personally crowned it by wearing this medal himself. The sinking of the Royal Mail Steamship *Leinster* is in truth Ireland's *Lusitania*. Five hundred murders, perpetrated in a moment of time, is the enemy's latest sample of "friendly" negotiation. Ireland's apostles of freedom, please note.

